# SPAD-based leaf nitrogen estimation is impacted by environmental factors and crop leaf characteristics

**DOI:** 10.1038/srep13389

**Published:** 2015-08-25

**Authors:** Dongliang Xiong, Jia Chen, Tingting Yu, Wanlin Gao, Xiaoxia Ling, Yong Li, Shaobing Peng, Jianliang Huang

**Affiliations:** 1 National Key Laboratory of Crop Genetic Improvement, MOA Key Laboratory of Crop Ecophysiology and Farming System in the Middle Reaches of the Yangtze River, College of Plant Science and Technology, Huazhong Agricultural University, Wuhan, Hubei 430070, China; 2 College of Plant Science and Technology, Huazhong Agricultural University, Wuhan, Hubei 430070, China; 3 Hubei Collaborative Innovation Center for Grain Industry, Yangtze University, Jingzhou, Hubei 434023, China

## Abstract

Chlorophyll meters are widely used to guide nitrogen (N) management by monitoring leaf N status in agricultural systems, but the effects of environmental factors and leaf characteristics on leaf N estimations are still unclear. In the present study, we estimated the relationships among SPAD readings, chlorophyll content and leaf N content per leaf area for seven species grown in multiple environments. There were similar relationships between SPAD readings and chlorophyll content per leaf area for the species groups, but the relationship between chlorophyll content and leaf N content per leaf area, and the relationship between SPAD readings and leaf N content per leaf area varied widely among the species groups. A significant impact of light-dependent chloroplast movement on SPAD readings was observed under low leaf N supplementation in both rice and soybean but not under high N supplementation. Furthermore, the allocation of leaf N to chlorophyll was strongly influenced by short-term changes in growth light. We demonstrate that the relationship between SPAD readings and leaf N content per leaf area is profoundly affected by environmental factors and leaf features of crop species, which should be accounted for when using a chlorophyll meter to guide N management in agricultural systems.

Crops are highly dependent on inputs of nitrogen (N) fertilizer to achieve optimum production in agricultural systems. Hence, excess N fertilizer is frequently supplied to obtain a high yield in most modern agricultural production systems. The increasing cost of chemical N fertilizer production, its energy requirement and numerous negative environmental effects such as water pollution^1^,^2^ and greenhouse gas emission^3^,^4^, have stimulated much research activity aiming to enhance the N use efficiency (NUE) of main crops. Improving the congruence between crop N demand and the N supply available from soil and applied fertilizer is one strategy to increase NUE in these systems^5^,^6^. For this purpose, early studies have focused primarily on soil-based strategies, such as identifying the most appropriate timing for split applications and optimizing fertilizer placement methods and fertilizer formulations^7^,^8^. However, those strategies have not improved the congruence between the N supply from applied fertilizer and crop demand because of the dynamic N requirements of crops.

Another approach to improving NUE involves plant-based strategies that rely on monitoring the N status of crops by measuring chlorophyll content per leaf area^9^,^10^,^11^,^12^. Chlorophyll, which is one of the most important chelates for plants, is capable of channeling the energy of sunlight into chemical energy through the process of photosynthesis. In addition to indicating plant nitrogen status, chlorophyll content is an important indicator of leaf senescence^13^, and it can also be altered in response to environmental stresses^14^. There are several methods for examining chlorophyll content. The extraction method, involving extraction of chlorophyll in a solvent followed by *in vitro* measurements with a spectrophotometer, is destructive, laborious, time consuming, and costly. However, chlorophyll meters, for example the SPAD-502 (Spectrum Technologies, Plainfield, Illinois, USA), provide a simple, quick, and nondestructive method for estimating leaf chlorophyll content. The SPAD meter has been widely used in both research and agricultural settings^9^,^10^,^11^,^12^,^15^,^16^.

SPAD readings are calculated based on two transmission values: the transmission of red light at 650 nm, which is absorbed by chlorophyll, and the transmission of infrared light at 940 nm, at which no chlorophyll absorption occurs. Using SPAD meter to assess leaf chlorophyll concentration has become common, but calibrating SPAD readings into direct units of chlorophyll concentration is still difficult, and an understanding of the relationship between these two parameters is necessary^17^. Numerous studies have estimated the relationship between SPAD readings and chlorophyll content per leaf area in different species. However, the relationship between SPAD readings and chlorophyll content per leaf area has been found to vary widely among species, in some cases even within a same species^18^,^19^,^20^. This variability is presumed to be due to variability of measurement conditions^21^ and to structural differences aomg the leaves that cause different light reflection and/or scattering effects. These results suggest that the relationship between SPAD readings and chlorophyll content per leaf area remains to be stablished.

Approximately 80% of leaf N is allocated to chloroplasts and approximately 50% of leaf N is invested in photosynthetic proteins in leaves. However, only 0.5–1.5% of leaf N is allocated to chlorophyll depending on the plant’s growth environment and species^22^,^23^. The increased amount of leaf N allocated to chlorophyll–protein complexes with decreasing irradiance has been observed in many species^23^,^24^. This pattern is consistent with the expected optimal pattern of nitrogen partitioning that maximizes the daily CO_2_ carbon gain of individual leaves^25^. Furthermore, the allocation ratio of leaf N to chlorophyll is affected by N supplementation conditions^26^. Knowing the effects of leaf characteristics and environmental factors on SPAD readings and the relationship between chlorophyll content and leaf N content per leaf area will be important questions when the SPAD-502 is used to guide N management practices in agriculture systems.

The objectives of this study were as follows: (1) to estimate the variations in SPAD readings and chlorophyll content per leaf area among species; (2) to identify the impacts of leaf features and light conditions on the relationships between SPAD values and chlorophyll content and leaf N content; and (3) to clarify the risks of relying on SPAD readings for N management.

## Results

### Relationship between SPAD and Chlorophyll content per leaf area

**Figure 1:**
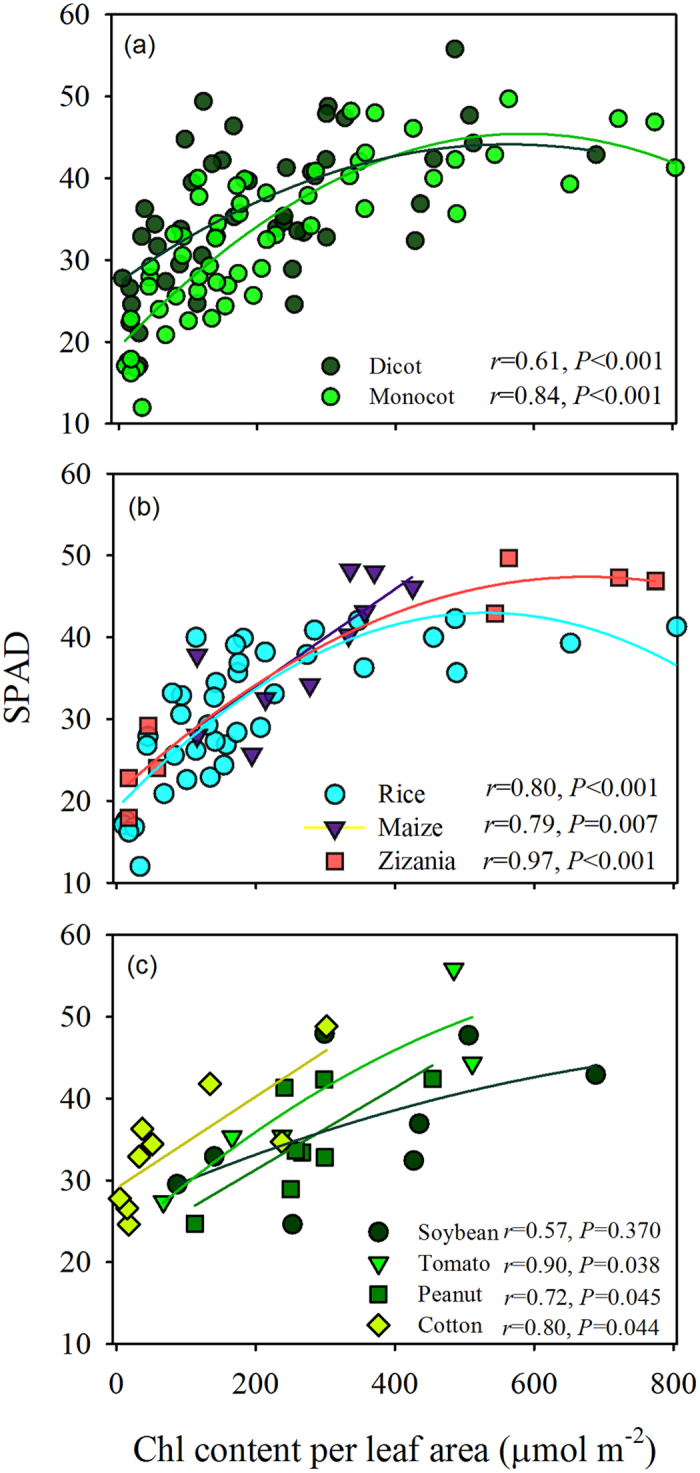
The relationship between SPAD values and chlorophyll content per leaf area for (**a**) dicot and monocot, (**b**) species of monocot, and (**c**) species of dicot.

**Figure 2:**
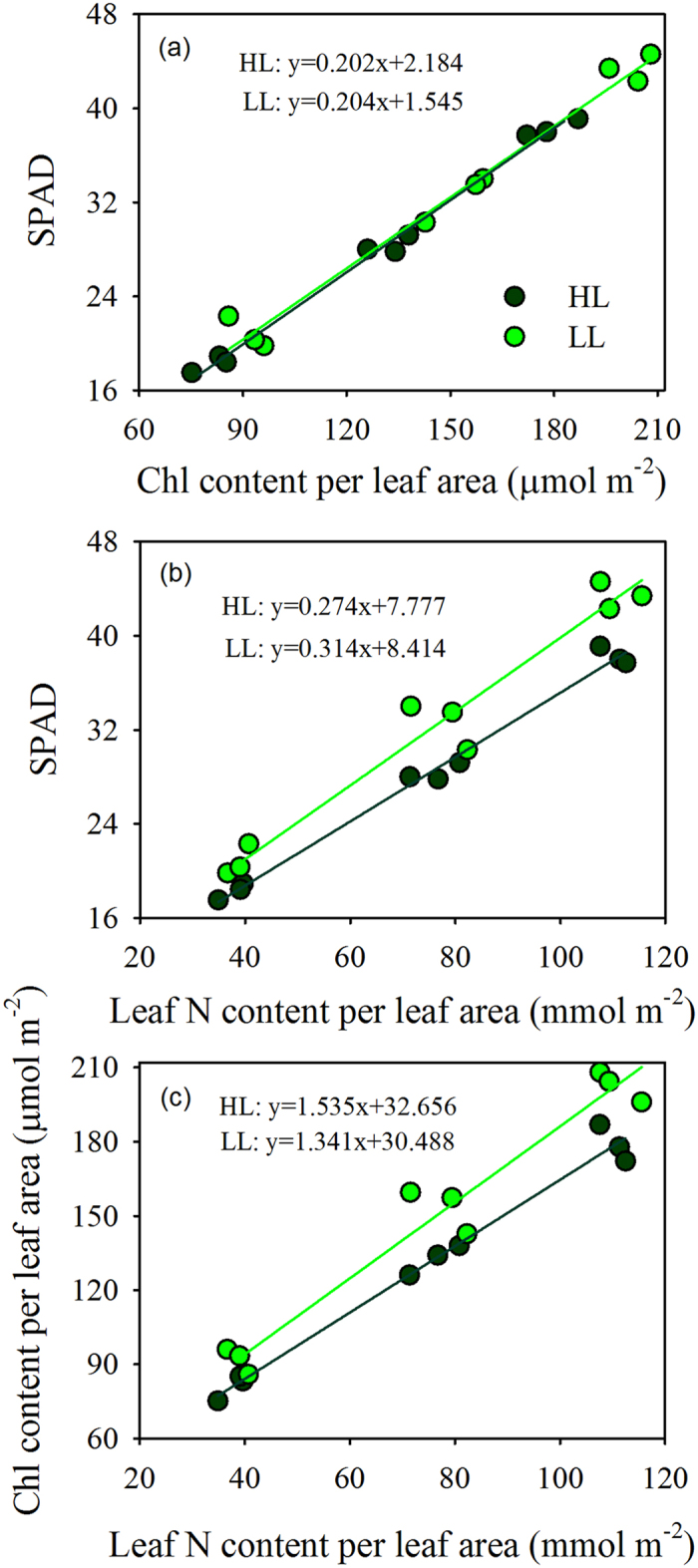
The relationships between (**a**) SPAD and chlorophyll content per leaf area, (**b**) SPAD and leaf N content per leaf area, and (**c**) chlorophyll content per leaf area and leaf N content per leaf area response to 7 days low light treatment in rice. HL, high light; LL, low light.

There was a close relationship between SPAD value and chlorophyll content per leaf area in both the monocot and dicot groups (Fig. 1). The relationships between SPAD and chlorophyll content per leaf area established from the mean of three monocot species and the mean of four dicot species were not significantly different. The seven species in this study exhibited a wide range of relationships between SPAD and chlorophyll content per leaf area. However, in the monocot group, rice, maize and Zizania showed similar relationships between SPAD and chlorophyll content per leaf area. In contrast, seven days of low light treatment had no effect on the relationships between SPAD and chlorophyll content per leaf area (Fig. 2a).

### Diurnal variation in SPAD readings

**Figure 3:**
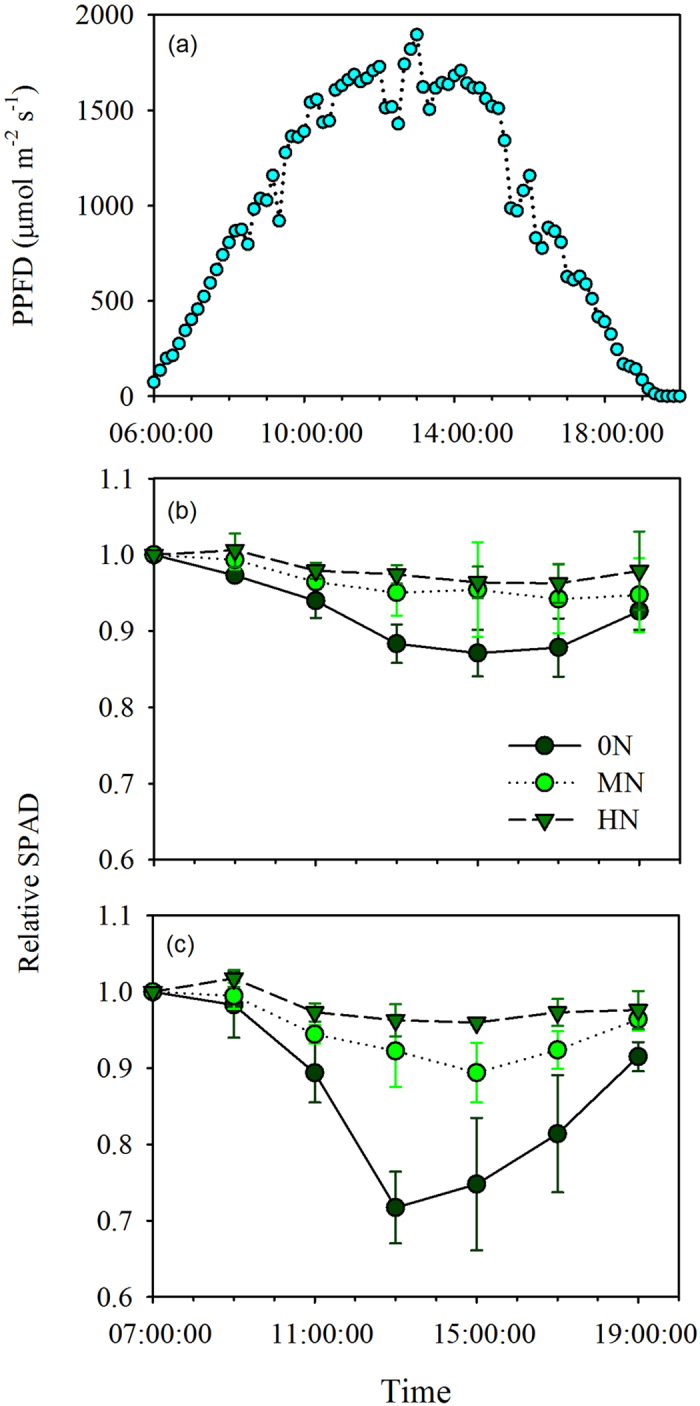
Relative SPAD (normalized by SPAD at 7:00) response to diurnal PPFD change. (**a**) The PPFD of measurement day, (**b**) rice, and (**c**) soybean. The data are shown as the mean ± SD of three pot replicates.

Diurnal variation in SPAD readings were dependent on the species and N supplementation (Fig. 3). For rice, there was no significant diurnal variation in SPAD readings from either high N or middle N supplementation, but the SPAD readings from 0 N supplementation were significantly lower at midday (Fig. 3b). For soybean, the SPAD readings from 0 N and middle N supplementation were significantly reduced at midday, and there was no significantly decreased with high N supplementation (Fig. 3c). There were maximal decreases of 13.0% and 28.2% for SPAD readings at midday with 0 N supplementation of rice and soybean, respectively (Fig. 3).

### Relationship between SPAD and leaf N content per leaf area

**Figure 4:**
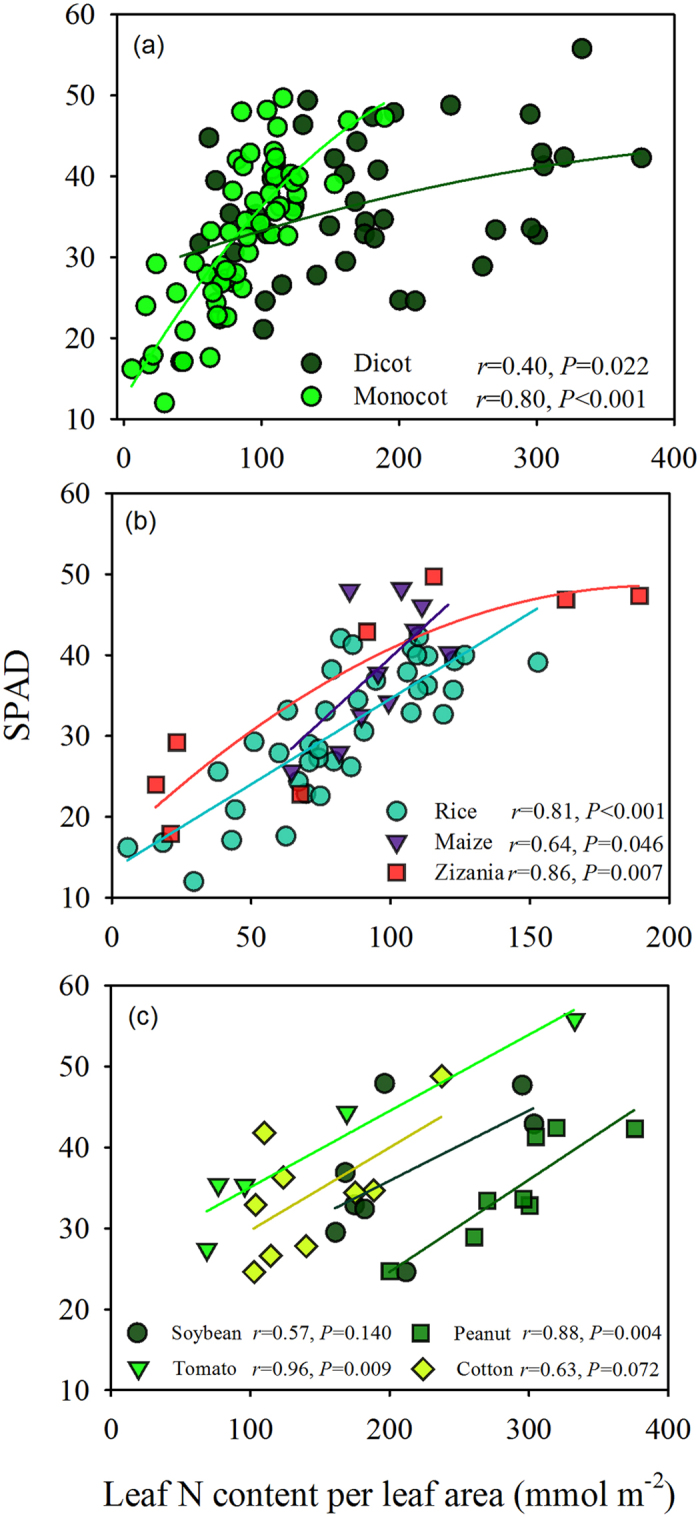
The relationship between SPAD value and leaf N content per leaf area for (**a**) dicots and monocots, (**b**) species of monocot, and (**c**) species of dicot. Values are means of 10 to 15 measurement points.

There was a close relationship between SPAD readings and leaf N content per leaf area. However, the relationship of SPAD readings to leaf N content per leaf area was significantly different between the monocot and dicot groups (Fig. 4a). In each group, the relationship was strongly dependent on the species (Fig. 4b,c). Unlike the relationship between SPAD readings and chlorophyll content per leaf area, the relationship between SPAD readings and leaf N content per leaf area was significantly affected by seven days of low light treatment (Fig. 2b).

### Relationship between leaf N and chlorophyll content per leaf area

**Figure 5:**
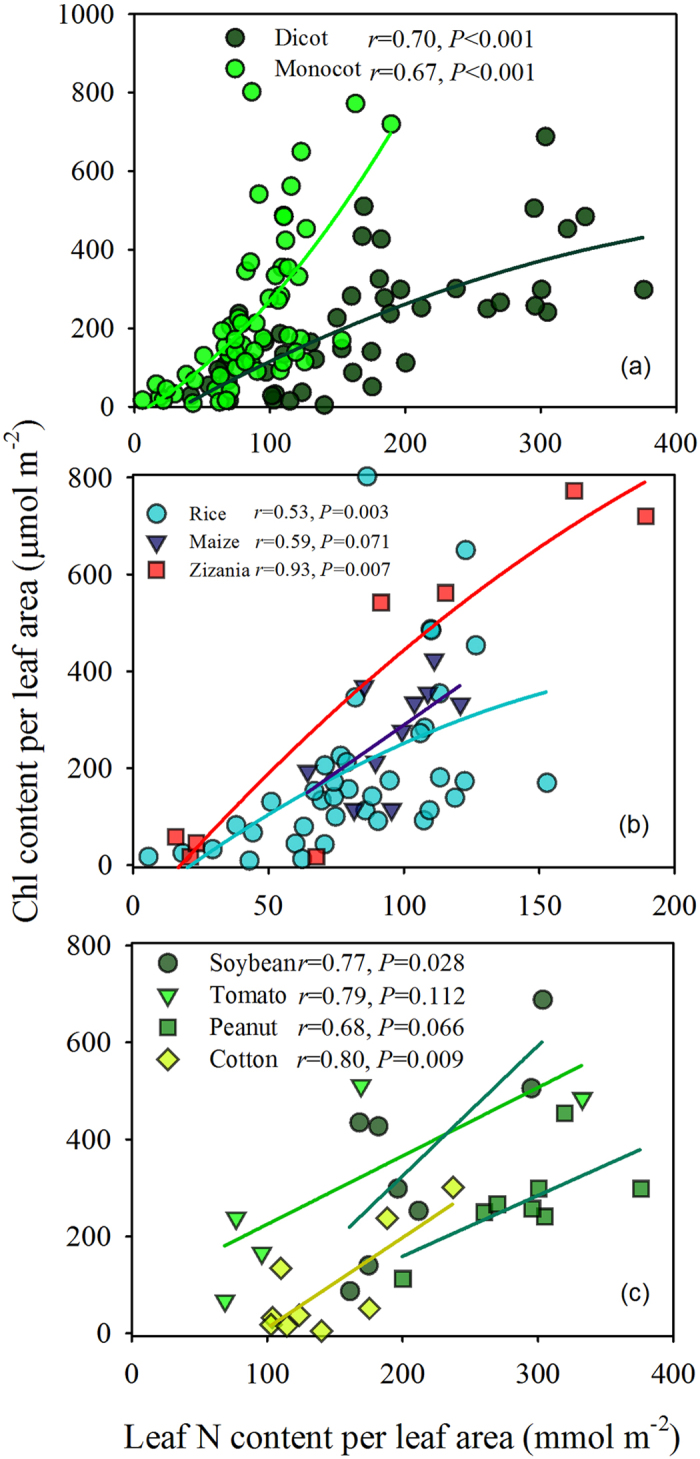
The relationship between chlorophyll content per leaf area and leaf N content per leaf area for (**a**) dicots and monocots, (**b**) species of monocot, and (**c**) species of dicot.

The chlorophyll content per leaf area increased with increasing N content per leaf area in both the monocot and dicot groups (Fig. 5a). However, the relationship between chlorophyll content and N content per leaf area was significantly different in the monocot and dicot groups. In each group, the relationship was strongly dependent on the species (Fig. 5b,c). Moreover, the relationship between chlorophyll content and N content per leaf area was significantly different under natural conditions and with seven days of low light treatment (Fig. 2c).

### Effects of N supplementations on leaf features

**Figure 6:**
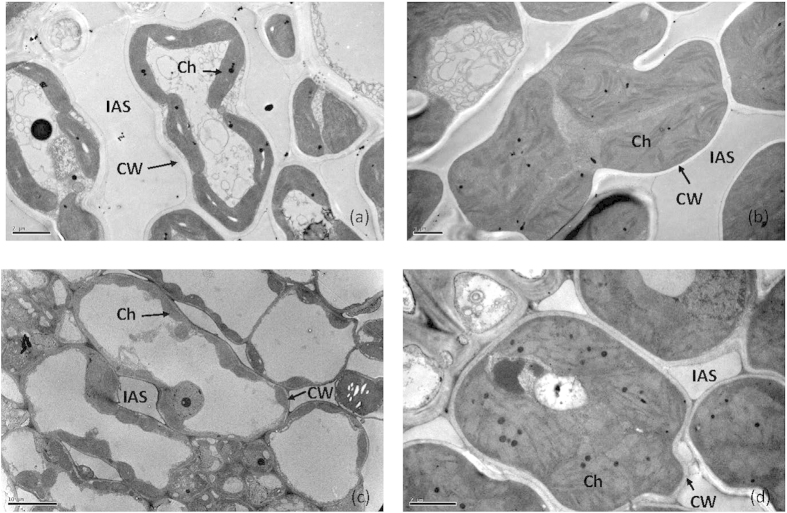
Transmission electron micrographs of armed chlorenchyma cells of rice at 0 N (**a**) and high N (**b**), and soybean at 0 N (**c**) and high N (**d**) supplement. IAS, intercellular air space; CW, cell wall; Ch, chloroplast.

A significant difference in chloroplast number per planar mesophyll cell between low and high N supplementation conditions was observed in rice, but not in soybean (Table 1). In addition to their number, the size of chloroplast was affected by N supplementation. In the present study, the planar area of chloroplast was profoundly enhanced by high N supplementation in both rice and soybean, resulting in a significant increase in chloroplast planar area per planar cell area (Table 1, Fig. 6).

## Discussion

### Leaf characteristics and environmental factors influencing SPAD readings

In most early studies, the relationship between SPAD readings and chlorophyll content per leaf area was fitted as a linear regression^11^,^12^. However, the results from the present study (Fig. 1) and other studies^19^,^27^,^28^ show that SPAD readings correlate non-linearly with chlorophyll content per leaf area. A photon reaching a leaf is absorbed, reflected or transmitted, and its fate is substantially affected by the distribution of chlorophyll within the leaf, which is determined by the structural organization of grana within chloroplasts, chloroplasts within cells, and cells within tissue layers^29^. A non-uniform distribution of chlorophyll in leaves may lead to the sieve and detour effects. In the sieve effect, which increases with increasing non-uniformity of chloroplasts, light passes through leaf tissue without encountering an absorber. The critical value of the path length for red light transmittance is based on the assumption that the path length can be calculated from the NIR transmittance. As chloroplast non-uniformity increases, the red light absorption rate decreases. The detour effect (light scattering) is caused by leaf reflectance at the reference NIR wavelength being higher than the leaf reflectance at the red chlorophyll absorption wavelength. Furthermore, the NIR wavelength can be absorbed by non-chlorophyll compounds^19^.

In past decades, many investigations have sought to determine how the relationship between SPAD and chlorophyll content per leaf area varies among species and growth habit groups. Cerovic, Masdoumier^30^ suggested that a significant difference exists between monocot and dicot species by measuring two monocot and two dicot species. However, in the present study, we investigated the relationship between SPAD and chlorophyll content per leaf area in three monocots and four dicots, and the results suggested that there is no significant difference between monocots and dicots (Fig. 1a), which was also suggested by Parry, Blonquist^19^. There were also no differences among the monocot species (Fig. 1b), but we could not derive a definite conclusion regarding the differences among dicots due to insufficient samples numbers (Fig. 1c), and this issue should be examined in the future. Identifying differences between C_3_ and C_4_ plants in the relationship of SPAD to chlorophyll content per leaf area is another important issue. In the present study, there was no difference between the C_4_ plant (maize) and two C_3_ plants (rice and Zizania). Our results suggest that different crops share a common relationship between SPAD and chlorophyll content per leaf area under standard measurement conditions.

Several studies have reported that SPAD readings are significantly affected by environmental light conditions due to light-dependent chloroplast movement^31^. In our study, diurnal changes in SPAD readings were found under low N supplementation conditions but not under high N supplementation conditions in both rice and soybean (Fig. 3). This difference is mainly due to enlarged chloroplasts occupying almost the entire cell space, which inhibited chloroplasts movement under high N conditions (Fig. 6). In rice, the chloroplasts covered most of the rice mesophyll cell periphery^32^,^33^ even under 0 N supplementation condition (Table 1, Fig. 6a), and this is one of possible reason for the milder diurnal changes of SPAD readings in rice than in soybean under low N supplementation conditions. Our results suggest that the effects of light-dependent movement on SPAD readings are related to both species and leaf N status.

### Leaf features and environmental factors influencing leaf N allocation to chlorophyll

Chlorophyll meter-based crop N managements regimes assume that the relationship between chlorophyll content and leaf N content per leaf area^34^ is stable. However, only a small fraction of leaf N is allocated to chlorophyll^35^, as confirmed in the present study (Fig. 5). The proportionality between chlorophyll content and leaf N content per leaf area may vary because nitrogen partitioning among photosynthetic proteins changes in response to light, nitrogen supplementation and among species^25^,^36^,^37^. In the present study, the relationship between chlorophyll content and N content per leaf area in monocots was significantly different from that in dicots. Interestingly, the proportion of leaf N allocated to chlorophyll increased with increasing leaf N content in monocots, but it decreased in dicots (Fig. 5), which may be attributable to their structural differences.

In addition to species differences, we observed that the allocation of total leaf N to chlorophyll was affected by light intensity (Fig. 2). This finding is consistent with the expected optimal pattern of nitrogen partitioning that maximizes the daily CO_2_ carbon gain of individual leaves. The ability to adjust the allocation leaf N to different pools of the photosynthetic machinery enhances the plants ability to adapt to different growth environments. These results indicate that the relationship between chlorophyll content and N content per leaf area varies among species, and it is also sensitive to the environment.

### Potential risks

Nitrogen is an essential nutrient for the growth of crops, and it is one of the most difficult nutrients to manage. When excessive N fertilizer is introduced to an agricultural system, crops are more susceptible to environmental stress and pest pressure, but with insufficient N fertilizer input, crop health and productivity suffer. A crucial aspect of achieving optimal N fertilizer management is to determine the dynamic N requirement of plants. Many N fertilizer managements regimes have been developed, including chlorophyll meter-based methods. The principle of chlorophyll meter-based method is that leaf N status is reflected in leaf greenness, which is reflected by values such as SPAD, CCI and NDVI. Based on these techniques, several N management strategies have been developed for rice^11^, wheat^38^,^39^, maize^12^ and cotton^40^. However, the achievements of these strategies differ greatly among studies and crop species.

**Figure 7:**
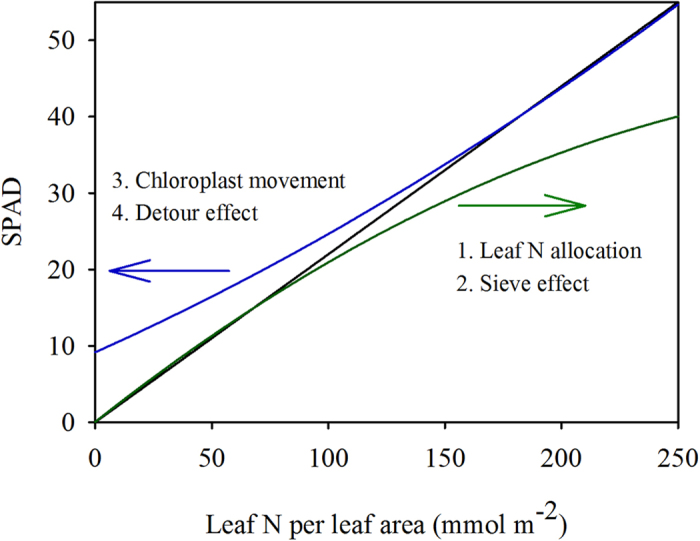
Summarized relationships between the SPAD values and leaf N content per leaf area in rice. The black line represents the theoretical relationship. The blue line represents the impacts of chloroplast movement and/or detour effect. The green line represents impacts of leaf N allocation and/or sieve effect.

The key step in SPAD-based N management regimes is to establish a threshold value of leaf N status for N application, which is represented by SPAD readings according to the known relationship between SPAD readings and leaf status. In this study, we found that the relationship between SPAD readings and leaf N content per leaf area varies among species, and it can be affected by leaf features and environmental factors. Our findings indicate that the threshold SPAD value for N application varies with the species and the environment. Based on our findings, the potential risks of SPAD reading-dependent nitrogen management regimes are summarized in Fig. 7. On one hand, leaf N status may be underestimated under relatively high leaf N status, because the SPAD readings are significantly impacted by sieve effects^19^,^41^, and the allocation of leaf N to chlorophyll is more susceptible to changes in environmental conditions, e.g. shading (Fig. 2). On the other hand, leaf N status may be overestimated under relatively low leaf N status because the SPAD readings are markedly affected by light-dependent chloroplast movements (Fig. 3) and detour effects^18^,^19^.

In conclusion, in this study, we provide evidence that the relationship between SPAD readings and N content per leaf area is profoundly affected by environmental factors and leaf features of crop species. The results suggest that the time of SPAD measurements, environmental irradiance and species must be taken into account to precisely monitor leaf N status with chlorophyll meters.

## Materials and Methods

### Experiment 1

To investigate the variation in the relationships among SPAD readings, chlorophyll content per leaf area and leaf N content per leaf area, leaves of multiple ages and with green color of variable intensity were measured and sampled from seven species (rice, maize, Zizania, soybean, tomato, peanut and cotton) grown in field and pot conditions. SPAD measurements were made with a SPAD-502 instrument between 7:00 and 9:00 am to minimize the potential effects of light intensity on chloroplast movement, with a SPAD-502. For monocot leaves, ten SPAD readings taken around the midpoint of each leaf, on one side of the midrib were averaged. For dicot leaves, ten to fifteen SPAD readings taken around the leaf edges were averaged. After the SPAD readings were recorded, the leaves were sampled on ice with plastic bags to measure area, chlorophyll content, dry weight, and leaf N content.

### Experiment 2

A pot experiment was conducted to investigate the effects of short-term shade on the relationships among SPAD readings, chlorophyll content per leaf area and leaf N content per leaf area. The rice cultivar Gangyou 118 was grown in 15.0 L pots with a density of three hills per pot and one seedling per hill. Each pot was filled with 13.0 kg soil, and P and K were applied as basal fertilizers at a rate of 1.95 g KH_2_PO_4_ per pot. Three N treatments (0 N: 0 g N, MN: 1.0 g N and HN: 2 g N per pot, urea was used) were set up in this experiment. Seedlings were grown outside and irrigated daily with water as required, and pests were controlled using chemical pesticides. At 60 days after germination, half of the pots in each treatments were moved to the shade equipment, which shaded natural light with semitransparent glass (inside PAR approximately half of that outside; data not shown). These SPAD measurements were also performed between 7:00 to 9:00 am. To avoid the effects of leaf ages on SPAD readings, the measurements were performed on new fully expanded leaves that adjacent to a similar leaf about to emerge. The average of ten SPAD readings around the midpoint of each leaf was taken, both before and seven days after the shade treatment. After recording the SPAD readings, the leaves were sampled on ice with plastic bags to measure area, chlorophyll content, dry weight, and leaf N content.

### Experiment 3

To investigate the impacts of light-dependent chloroplast movement on SPAD readings, a pot experiment was conducted. The rice cultivar Huanghuazhan and a soybean cultivar Zhongdou 8 were grown in 15.0 L pots with a density of three hills per pot and one seedling per hill. Fertilizer application, N treatment, and pest management were the same as in experiment 2 and the plants were well watered to avoid drought stress or waterlogging (for soybean). The measurements were conducted on marked, new fully expanded leaves at 46 days after germination. SPAD readings were taken every two hours from 7:00 am to 7:00 pm. For rice leaves, ten SPAD readings taken around the midpoint of each leaf, on one side of the midrib were averaged. For soybean leaves, the average of fifteen (five point on each leaflets) SPAD readings taken around leaf edges was used. Chloroplast movements is substantially affected by light conditions; chloroplasts move away from strong light (avoidance response) to avoid photodamage, and they move toward weak light (accumulation response) to receive more light for photosynthesis. Because of the uniformity of light (darkness) around leaves during the night, chloroplasts should be arranged uniformly inside the leaves, which is the perfect condition for SPAD measurements (Fig. 3). Based on this reasoning, the SPAD readings of each leaf were normalized to the SPAD reading at 7:00 am (relative SPAD = [SPAD reading/SPAD reading at 7:00 am]). Due to changes in cloud cover, the PPFD during the midday is also highly variable. To avoid the potential effects of moving cloud cover, the leaves were unshaded at least 30 min before the measurements were performed. The leaves marked for of 0N and HN treatments were sampled for transmission electron microscope (TEM) analysis on the morning of the next day.

### Leaf area, chlorophyll and N measurement

Leaf samples were taken to the lab on ice. After photo scanning, each sample leaf was cut in to small sections and then separated into two parts for chlorophyll and leaf N content measurement. The fresh samples were weighed with an electronic balance (CP2102, Ohaus Corporation). Absolute chlorophyll concentration measurements were conducted using a spectrophotometer (UV2102, Unico, China) and 95% (v/v) alcohol extracts of leaf tissue. The samples for leaf N measurement were oven-dried at 80 °C to constant weight and digested by using the micro-Kjeldahl method, after which the N concentration was measured with a discrete wet chemistry analyzer (SmartChem® 200, AMS-Westco, Italy). The leaf area was measured with Image J software (the National Institutes of Health).

### TEM analysis

Small leaf discs about 4.0 × 1.2 mm were removed from the middle of new fully expanded leaves (avoiding the midribs). The leaf sections were infiltrated in a syringe with a fixative of 2.5% glutaric aldehyde in 0.1 M phosphate buffer (pH = 7.6) at 4 °C, and post-fixed in 2% buffered osmium tetroxide at 20 °C for 2 h. Three leaves from each treatments were analyzed. Ultrathin leaf cross sections were stained with 4% (w/v) uranyl acetate followed by 2% (w/v) lead citrate. An h-7650 transmission electron microscopes (Hitachi—Science & Technology, Japan) and Soft Imaging System software were used for observation and photography. Then, the images were analyzed by using image J software (the National Institutes of Health).

### Statistical analysis

Analyses of covariance (ANCOVA) were conducted to differentiate between the effects of the N treatments on leaf structural parameters using SAS 9.2 (SAS Institute Inc.) in experiment 3. The mean values were compared using one way analysis of variance with a Tukey test (P < 0.05). Regression analyses were performed to test the correlations between parameters using SigmaPlot 12.5 (SPSS Inc., Chicago, IL, USA). All regressions were fitted by both linear and power models, and the model with the higher regression coefficient was selected. Regression coefficients were tested by the Shapiro-Wilk method and significance is shown for *P* < 0.05.

## Additional Information

**How to cite this article**: Xiong, D. *et al*. SPAD-based leaf nitrogen estimation is impacted by environmental factors and crop leaf characteristics. *Sci. Rep*. **5**, 13389; doi: 10.1038/srep13389 (2015).

## Figures and Tables

**Table 1:** Properties of the mesophyll cell chloroplasts in rice and soybean plants with different N treatments.

Species	Rice		Soybean	
N treatment	LN	HN	LN	HN
Chloroplast number per planar cell	7.25 ± 1.48 a	5.80 ± 0.51 b	5.92 ± 0.67 b	6.03 ± 0.43 b
Planar area per chloroplast, μm^2^	5.36 ± 1.01 c	18.28 ± 2.95 a	2.31 ± 0.36 d	12.39 ± 2.12 b
Chloroplast planar area per planar cell area, %	49.2 ± 5.0 b	91.5 ± 4.8 a	23.6 ± 4.7 c	92.1 ± 1.9 a

Values were obtained from transmission electron microscopy images of transverse sections of leaves. Area measurements are from planar views through leaf sections. Mean ± SE (n = 12, three biological repeats); mean differences were tested using one-way analysis of variance with a Tukey test. Statistical groups are indicated by letters at P < 0.05.
